# Interplay between Zika Virus and Peroxisomes during Infection

**DOI:** 10.3390/cells8070725

**Published:** 2019-07-15

**Authors:** Cheung Pang Wong, Zaikun Xu, Shangmei Hou, Daniel Limonta, Anil Kumar, Christopher Power, Tom C. Hobman

**Affiliations:** 1 Department of Medical Microbiology & Immunology, University of Alberta, Edmonton, AB T6G 2E1, Canada; 2 Department of Cell Biology, University of Alberta, Edmonton, AB T6G 2H7, Canada; 3 Department of Medicine, University of Alberta, Edmonton, AB T6G 2E1, Canada; 4 Women & Children’s Health Research Institute, University of Alberta, Edmonton, AB T6G 1C9, Canada; 5 Li Ka Shing Institute of Virology, University of Alberta, Edmonton, AB T6G 2E1, Canada

**Keywords:** Zika virus, peroxisomes, innate immune response, interferon, astrocytes, fetal brain

## Abstract

Zika virus (ZIKV) has emerged as an important human pathogen that can cause congenital defects in the fetus and neurological conditions in adults. The interferon (IFN) system has proven crucial in restricting ZIKV replication and pathogenesis. The canonical IFN response is triggered by the detection of viral RNA through RIG-I like receptors followed by activation of the adaptor protein MAVS on mitochondrial membranes. Recent studies have shown that a second organelle, peroxisomes, also function as a signaling platforms for the IFN response. Here, we investigated how ZIKV infection affects peroxisome biogenesis and antiviral signaling. We show that ZIKV infection depletes peroxisomes in human fetal astrocytes, a brain cell type that can support persistent infection. The peroxisome biogenesis factor PEX11B was shown to inhibit ZIKV replication, likely by increasing peroxisome numbers and enhancing downstream IFN-dependent antiviral signaling. Given that peroxisomes play critical roles in brain development and nerve function, our studies provide important insights into the roles of peroxisomes in regulating ZIKV infection and potentially neuropathogenesis.

## 1. Introduction

Zika virus (ZIKV) is a mosquito-borne flavivirus whose genome consists of a positive sense single-stranded RNA that encodes three structural proteins (capsid, pre-membrane/membrane and envelope proteins) and seven non-structural proteins (NS1, NS2A, NS2B, NS3, NS4A, NS4B, and NS5) [1,2]. Until recently, ZIKV circulated primarily within Africa and Asia, but the large number of microcephaly cases and other neurological disorders associated with the 2015/2016 pandemic constituted a public health emergency of international concern [3,4]. While intensive research efforts have led to multiple promising vaccine candidates, there are currently no prophylactic or therapeutic antivirals available for this pathogen.

The interferon (IFN) system, a crucial arm of the innate immune system, has been shown to play a role in restricting ZIKV replication and pathogenesis [5,6,7]. The canonical IFN response is initiated following the detection of viral genomes/transcripts by RNA-sensing helicases such as RIG-I and MDA5 [8,9]. After binding viral RNA, these helicases interact with the adaptor protein MAVS, the bulk of which is localized on mitochondria. The activation of MAVS leads to the phosphorylation of the antiviral transcription factors IFN regulatory factor 3 (IRF3) and NF-κB, followed by transcription of *IFNβ* and *IFNλ* genes [10]. Secreted type I and III IFNs bind to receptors on the cell surface that signal through the JAK/STAT pathway to induce the transcription of IFN-stimulated genes (ISGs), resulting in an antiviral state [11,12]. However, many viruses, including flaviviruses, are known to deploy an array of counter-measures to suppress IFN induction and downstream antiviral signaling [13,14]. 

In addition to mitochondria, peroxisomes, which are membrane-bound organelles that have well characterized functions in lipid metabolism and regulation of reactive oxygen species [15,16], have recently been shown to play critical roles in antiviral defense. Specifically, activation of MAVS on peroxisomal as well as mitochondrial membranes appears to be important for IFN induction and signaling [17,18,19]. Evidence indicating that viruses disrupt peroxisome biogenesis began to emerge shortly after, further supporting the importance of peroxisomes in antiviral defense. First, we showed that in cells infected with West Nile (WNV) or Dengue (DENV) viruses, a critical peroxisome biogenesis factor, PEX19, is selectively degraded [20]. This process, which involves the capsid proteins of WNV and DENV, results in reduced levels of peroxisomes and a dampened type III IFN response [20]. Subsequently, it was reported that the NS3-4A protease of hepatitis C virus cleaves MAVS localized on peroxisomes and mitochondria [18,21], whereas the nsp1 protein of porcine diarrhea virus reduces type III IFN induction, in part by reducing peroxisome pools via an unknown mechanism [22]. Finally, human immunodeficiency virus-1 (HIV-1) infection was shown to downregulate peroxisomes by upregulating cellular microRNAs that inhibit the expression of peroxisome biogenesis factors such as PEX2, PEX7, PEX11 and PEX13 [23]. 

More recently, it was reported that the infection of Vero cells with ZIKV results in a 12% decrease in peroxisome density as well as a 50% loss of the peroxisomal membrane protein PMP70 [24]. It was hypothesized that during ZIKV infection, peroxisomes are consumed and, accordingly, that these organelles are actually required for ZIKV replication. However, this notion contrasts with mounting evidence supporting an antiviral role for peroxisomes [17,18,19,20,21,22]. 

Here, we investigated the interplay between ZIKV infection and peroxisomes in primary human fetal astrocytes (HFAs), the most abundant cell type in the brain and potentially a cellular reservoir for ZIKV [25]. Iinfection of HFAs resulted in a dramatic reduction in peroxisomes, regardless of the type of ZIKV strain employed. PEX11B, a biogenesis factor that induces peroxisome proliferation, was found to be a restriction factor for ZIKV. Elevated expression of PEX11B was associated with increased levels of MAVS and enhanced IFN induction and downstream signaling. As peroxisomes are critical for brain development and function [26,27], it is tempting to speculate that the loss of these organelles in HFAs may play a role in the neurological deficits associated with in utero ZIKV infection.

## 2. Materials and Methods

### 2.1. Cells and Virus Infection 

A549, HEK293T, Vero and U251 cells were purchased from the American Type Culture Collection (Manassas, VA, USA). The cells were cultured in Dulbecco’s modified Eagle’s medium (DMEM; Gibco; Waltham, MA, USA) supplemented with 100 U/mL penicillin and streptomycin, 1 mM 4-(2-hydroxyethyl)-1-piperazineethanesulfonic acid (HEPES)(Gibco; Waltham, MA, USA), 2 mM glutamine (Gibco; Waltham, MA, USA), 10% heat-inactivated fetal bovine serum (FBS; Gibco; Waltham, MA, USA) at 37 °C in 5% CO_2_. Primary human fetal astrocytes (HFAs) were prepared as previously described [28] from 15–19 week aborted fetuses with written consent approved under the protocol 1420 by the University of Alberta Human Research Ethics Board (Biomedical). HFAs were grown in Minimum Essential Media (MEM) (1 g/L Glucose, 15 mM HEPES, Gibco; Waltham, MA, USA) supplemented with 10% FBS, L-glutamine, MEM non-essential amino acids, sodium pyruvate, and 1 g/mL glucose at 37 °C in 5% CO2. PLCal and PRVABC59 strains of ZIKV were kindly provided by Dr. David Safronetz (Public Health Agency of Canada). The Zika virus (strain H/PF/2013, French Polynesia) was kindly provided by Dr. Michael Diamond (Washington University School of Medicine, St. Louis, MO, USA). The Zika virus (strain MR766) was generated from a molecular clone [29] kindly provided by Dr. Matthew J. Evans (Icahn School of Medicine at Mount Sinai, New York, NY, USA). All virus manipulations were performed according to level-2 containment procedures. Virus stocks were generated in C6/36 cells and titrated by plaque assay using Vero cells.

### 2.2. Plasmids and Transfection 

A triple FLAG^®^ (DYKDDDDK)-tagged ZIKV capsid expression construct was generated by polymerase chain reaction (PCR) using a mammalian expression vector encoding the capsid protein [28] as template. The resulting PCR product was then cloned into pcDNA 3.1(-) plasmid using the restriction sites NheI and XhoI. pCMV3-PEX11B was purchased from Sino Biological Inc. Oligonucleotide primers were designed to amplify the desired PEX11B sequence and introduce a myc epitope tag cassette into the 5′ end of the cDNA. The resulting PCR product was then cloned into the lentiviral vector pTRIP-MCS-IRES-AcGFP [30]. All oligonucleotide primers used in this study are shown in Table S1.

For indirect immunofluorescence analysis in U251 cells, transfection of the appropriate expression plasmids was performed using Lipofectamine 2000 (Invitrogen; Carlsbad, CA). Poly(I:C) (Sigma-Aldrich; St. Louis, MO, USA) was transfected into cells using TransIT-LT1 (Mirus Bio; Madison, WI, USA). 

### 2.3. Production of Lentiviral Particles and Transduction of Cells

Pseudotyped lentiviruses were recovered from the media of HEK293T cells transfected with pTRIP-AcGFP plasmids encoding flavivirus capsids or PEX11B, and titered as described [30]. Transduction of U251 cells with recombinant lentiviruses was performed in DMEM containing 3% FBS and 5 μg/mL polybrene for 48 h in 12-well plates.

### 2.4. Antibodies

Primary antibodies were from the following sources: Mouse monoclonal antibodies against the peroxisomal membrane protein PMP70 and FLAG epitope from Sigma (St. Louis, MO, USA); mouse monoclonal against beta-actin, goat polyclonal antibody against GFP and rabbit polyclonal antibodies to PEX3, PEX7, PEX11B, PEX13, PEX19 and catalase from Abcam (Cambridge, MA, USA); rabbit polyclonal antibody to the tripeptide SKL was produced as described in [23]; goat polyclonal antibody to ZIKV NS5 was produced and purified as described in [31]; mouse monoclonal antibody against MAVS was from Santa Cruz Biotechnology (Santa Cruz, CA, USA); mouse monoclonal antibody against myc from Millipore (Burlington, MA, USA). Donkey anti-mouse IgG conjugated to Alexa Fluor 680, goat anti-rabbit IgG conjugated to Alexa Fluor 680, donkey anti-mouse IgG conjugated to Alexa Fluor 488, donkey anti-goat IgG conjugated to Alexa Fluor 680, chicken anti-goat IgG conjugated to Alexa Fluor 647, and donkey anti-rabbit IgG conjugated to Alexa Fluor 546 were purchased from Invitrogen (Carlsbad, CA, USA).

### 2.5. Immunoblotting

HFAs or U251 cells collected at designated time points post-infection were washed three times with phosphate-buffer saline (PBS) before lysing with sodium dodecyl sulfate (SDS) Sample Buffer containing β-mercaptoethanol (2%) and 1 unit of Benzonase (Millipore; Burlington, MA, USA) per sample. Proteins in the samples were denatured at 98 °C for 10 min, separated by SDS-PAGE and then transferred to polyvinylidene difluoride membranes for immunoblotting as described [31]. Proteins on the membranes were imaged and analyzed using an Odyssey^®^ CLx Imaging System (LI-COR Biosciences; Lincoln, NE, USA). Relative levels of MAVS, PMP70, PEX3, PEX7, PEX11B, PEX13, PEX19, and catalase (normalized to actin) were determined using Odyssey Infrared Imaging System 1.2 Version software.

### 2.6. Quantitative Real-Time PCR (qRT-PCR)

Total RNA from HFAs and U251 cells was isolated using the RNA NucleoSpin Kit (Machery Nagel; Bethlehem, PA, USA) and reverse transcribed with random primers (Invitrogen; Carlsbad, CA, USA) and the Improm-II reverse transcriptase system (Promega; Madison, WI, USA) at 42 °C for 2 h. The resulting cDNAs were mixed with the appropriate primers (Integrated DNA Technologies; Coralville, IA) and PerfeCTa SYBR Green SuperMix Low 6-Carboxy-X-Rhodamine (ROX) (Quanta Biosciences; Beverly, MA, USA) and then amplified for 40 cycles (30 s at 94 °C, 40 s at 55 °C and 20 s at 68 °C) in a CFX96 Touch™ Real-Time PCR Detection System. The gene targets and primers used are listed in (Table S2). The ΔCT values were calculated using β-actin mRNA as the internal control. The ΔΔCT values were determined using control samples as the reference value. Relative levels of mRNAs were calculated using the formula 2(−ΔΔCT) [32]. 

### 2.7. Co-Immunoprecipitation and Immunoblotting

U251 cells (1 × 10^6^), seeded the day before into 10 cm dishes, were infected with PRVABC59 strain of ZIKV (MOI = 1). At 48 h post-infection, cells were washed three times with PBS before lysing with NP-40 lysis buffer (150 mM NaCl, 2 mM ethylenediaminetetraacetic acid (EDTA), 1% Nonidet P-40, 50 mM Tris-HCl (pH 7.4), 1 mM dithiothreitol) containing CompleteTM protease inhibitors (Roche, Mannheim, Germany) on ice for 30 min. Lysates were clarified at 16,000 g for 20 min in a microcentrifuge at 4 °C. Small aliquots of the clarified lysates were kept for loading controls. The remaining lysates were treated with 20 μg/mL RNase A (Roche; Mannheim, Germany) for 1 h on ice, precleared with protein G-Sepharose beads (Sigma Aldrich; St. Louis, MO, USA) for 1 h at 4 °C before sequential incubation with antibodies overnight and then protein G-Sepharose beads for 2 h at 4 °C. Rabbit IgG was used in parallel as a negative control. Immunoprecipitates were washed three times with NP-40 lysis buffer before the bound proteins were eluted by boiling in SDS Sample buffer. Proteins were separated by SDS-PAGE and transferred to (PVDF) membranes for immunoblotting.

### 2.8. Cell Viability Assay

Cells were harvested in PBS and processed for cell viability assays using a CellTiter-Glo^®^ Luminescent Cell Viability Assay kit (Promega; Madison, WI, USA). 

### 2.9. Confocal Microscopy 

HFAs or U251 cells on coverslips were fixed for 15 min at room temperature with 4% electron microscopy grade paraformaldehyde (Electron Microscope Sciences; Hatfield, PA) in PBS. Samples were washed three times in PBS and incubated in blocking buffer (0.2% Triton X-100 (VWR Internationals; Radnor, PA, USA) and 3% bovine serum albumin (BSA; Sigma Aldrich; St. Louis, MO, USA) in PBS) at room temperature for 1.5 h. Incubations with primary antibodies diluted (1:1000) in blocking buffer (3% BSA and PBS) were carried out at room temperature for 2 h, followed by three washes in PBS containing 0.1% BSA. Samples were then incubated with secondary antibodies in Blocking buffer for 1 h at room temperature, followed by three washes in PBS containing 0.1% BSA. The secondary antibodies were Alexa Fluor 488 donkey anti-mouse, Alexa Fluor 546 donkey anti-rabbit, and Alexa Fluor 647 chicken anti-goat (Invitrogen; Carlsbad, CA, USA). Prior to mounting, samples were incubated with 5 μg/mL DAPI (4’,6-diamidino-2-phenylindole) for 5 min at room temperature before washing in PBS containing 0.1% BSA. Coverslips were mounted onto microscope slides using ProLong Gold antifade reagent with DAPI (Invitrogen; Carlsbad, CA, USA). Samples were examined using an Olympus 1 × 81 spinning disk confocal microscope equipped with a 60×/1.42 oil PlanApo N objective. Confocal images were acquired and processed using Volocity 6.2.1 software.

### 2.10. Quantification of Peroxisomes

*Z*-stack images acquired using a confocal microscope were exported from Volocity 6.2.1 as an OEM.tiff file. The exported images were then processed using Imaris 7.2.3 software (Bitplane, Concord, MA, USA). Peroxisomes within polygonal areas that excluded the nucleus were quantified (quality and voxel). Within the selected regions, the absolute intensity of the peroxisomes was determined and then entered into a Microsoft Excel spreadsheet. The data were then analyzed using student’s *t*-test. In each cell, peroxisomes were selected based on the absolute pixel intensity in the corresponding channel, and their numbers were then determined. Only those SKL/PMP70-positive structures with volumes between 0.001 and 0.05 μm^3^ were included for measurement.

### 2.11. Statistical Analyses

A paired Student’s *t*-test was used for pair-wise statistical comparison. The mean ± standard error of the mean is shown in all bar and line graphs. All statistical analyses were performed using Microsoft Excel software. 

## 3. Results

### 3.1. ZIKV Infection Decreases the Pool of Peroxisomes in Primary HFAs and U251 Cells

Given the important roles of peroxisomes in antiviral defense and brain development, we investigated how ZIKV infection of the most abundant cell type in the fetal brain affects these organelles. Primary HFAs were infected with four different ZIKV strains including two pandemic strains of Asian lineage; one isolated during the 2015/2016 outbreak in South America (PRVABC59) and one isolated from an outbreak in French Polynesia in 2013 (H/PF/2013). The other two strains included a third contemporary Asian strain (PLCal) isolated from a returning Canadian traveler [33] and the prototype African strain MR766. The effects of ZIKV infection on peroxisomes and peroxisomal proteins in HFAs were assessed by confocal microscopy analysis and immunoblotting, respectively.

**Figure 1:**
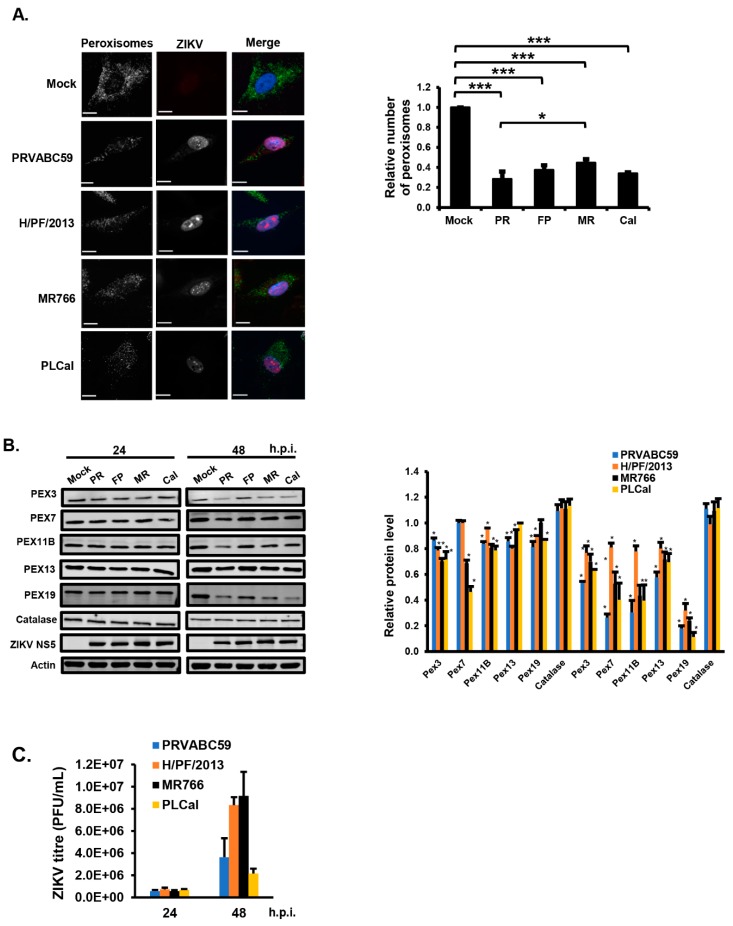
Zika virus (ZIKV) infection decreases the pool of peroxisomes in primary human fetal astrocytes (HFAs). (**A**) HFAs were infected at the multiplicity of infection (MOI) of 3) with ZIKV (PRVABC59 (PR), H/PF/2013 (FP), MR766 (MR) or PLCal (Cal) strains) for 48 h and then processed for confocal microscopy. Peroxisomes were detected using a mouse monoclonal to PMP70 and donkey anti-mouse IgG conjugated to Alexa Fluor 488. Infected cells were detected using a goat polyclonal antibody to ZIKV NS5 and chicken anti-goat IgG conjugated to Alexa Fluor 647. Nuclei were stained with DAPI (4’,6-diamidino-2-phenylindole). Images were obtained using a spinning disc confocal microscope. The number of peroxisomes in mock- and ZIKV-infected cells were determined using Volocity image analysis software. Averages were calculated from three independent experiments, in which a minimum of 20 cells for each sample were analyzed. The average number of peroxisomes in mock-treated cells was normalized to 1.0. Bars represent standard error of the mean. *** *p* < 0.001, * *p* < 0.05. (**B**) HFAs were infected with ZIKV strains (MOI = 5) for the indicated time periods, after which total cell lysates were processed for immunoblot analyses with antibodies to catalase, PEX3, PEX7, PEX11B, PEX13, PEX19, ZIKV NS5 and actin. The relative levels of peroxisomal proteins (compared to actin) from three independent experiments were averaged and plotted. The average levels of peroxisomal proteins in mock-infected cells were normalized to 1.0. Error bars represent standard error of the mean. * *p* < 0.05. (**C**) HFAs were infected with ZIKV (MOI = 1) for the indicated time periods, after which the viral titers in the media were determined by plaque assay. The data are averaged from the results of three independent experiments. Bars represent standard error of the mean.

Data in Figure 1A show that depending upon the viral strain, the numbers of peroxisomes as identified by staining with anti-PMP70 antibodies were reduced by 60–70% at 48-h post-infection. To rule out the possibility that the apparent loss of peroxisomes in ZIKV-infected HFAs was not due to decreased expression of PMP70 alone, the steady levels of other peroxisome-associated proteins were assessed by immunoblotting at 24- and 48-h post-infection. This included PEX3, PEX7, PEX11B, PEX13 and PEX19, all of which are critical for peroxisome biogenesis. The loss of multiple PEX proteins was evident at 24-h post-infection; however, by 48-h, the reduction in PEX3, PEX7, PEX11B, PEX13 and PEX19 was much more pronounced (Figure 1B). PEX19 was most sensitive to viral infection as its levels were decreased by as much as 90% depending upon the infecting ZIKV strain. Unlike peroxisome biogenesis factors, levels of the peroxisomal matrix protein catalase were not affected by ZIKV infection (Figure 1B). This was not unexpected, however, as a previous study showed that unlike many other cellular proteins that are degraded when not targeted to their proper location, catalase is quite stable in the absence of peroxisomes [23]. Infection with PRVABC59 and PLCal strains was associated with the greatest loss of PEX proteins from HFAs. This did not appear to be due to more robust replication as the infection of HFAs with PRVABC59, which consistently resulted in slightly lower titers than the other three strains (Figure 1C), generally had the greatest effect on peroxisomes (Figure 1A,B). 

**Figure 2:**
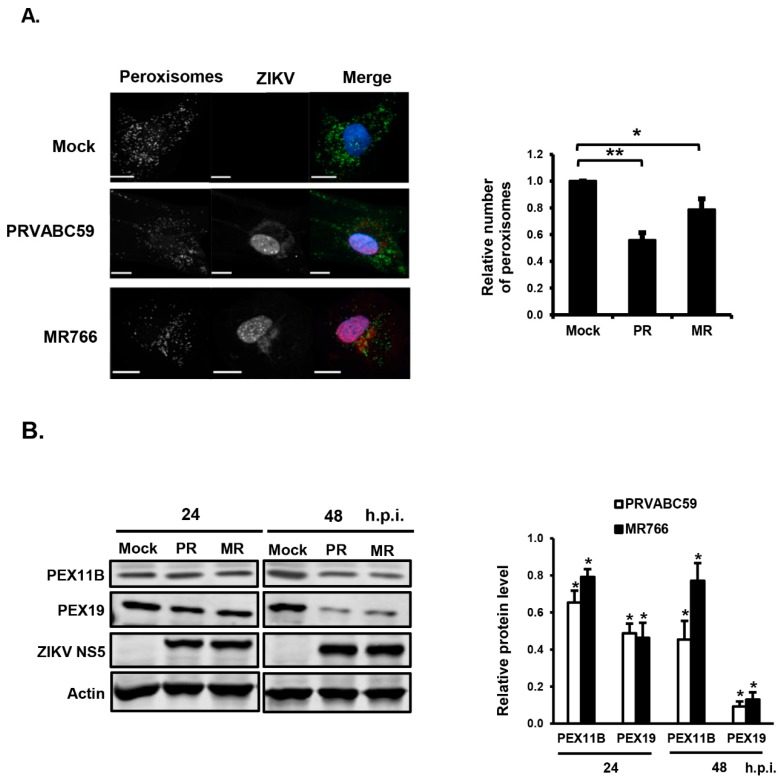
ZIKV infection decreases the number of peroxisomes in U251 cells. (**A**) U251 cells were infected with ZIKV PRVABC59 (PR) or MR766 (MR) using MOI = 3 for 48 h and then processed for confocal microscopy. Peroxisomes were detected using a mouse monoclonal to PMP70 and donkey anti-mouse IgG conjugated to Alexa Fluor 488. Infected cells were detected using a goat polyclonal antibody to ZIKV NS5 and chicken anti-goat IgG conjugated to Alexa Fluor 647. Nuclei were stained with DAPI. Images were obtained using a spinning disc confocal microscope. The number of peroxisomes in mock- and ZIKV-infected cells were determined using Volocity image analysis software. Averages were calculated from three independent experiments in which a minimum of 20 cells for each sample were analyzed. The average number of peroxisomes in mock-treated cells was normalized to 1.0. Bars represent standard error of the mean. ** *p <* 0.01, * *p* < 0.05. (**B**) U251 cells were infected with ZIKV PRVABC59 or MR766 (MOI = 5) for the indicated time periods, after which total cell lysates were processed for immunoblot analyses with antibodies to PEX11B, PEX19, ZIKV NS5 and actin. The relative levels of peroxisomal proteins (compared to actin) from three independent experiments were averaged and plotted. The average levels of peroxisomal proteins in mock-infected cells were normalized to 1.0. Error bars represent standard error of the mean. * *p* < 0.05. h.p.i.=hours post-infection.

Given that primary HFAs have a finite lifespan and limited expansion capacity, we assessed whether the human astrocytoma U251 cell line could be used to further examine the interplay between ZIKV infection and peroxisome biogenesis. Similar to HFAs, the infection of U251 cells with PRVABC59 or MR766 strains resulted in a significant loss of peroxisomes and peroxisome biogenesis factors (Figure 2A,B). Moreover, the infection of U251 cells with the pandemic strain PRVABC59 resulted in a greater loss of peroxisomes than MR766.

### 3.2. ZIKV Capsid Protein Binds PEX19 and Induces Loss of Peroxisomes

**Figure 3:**
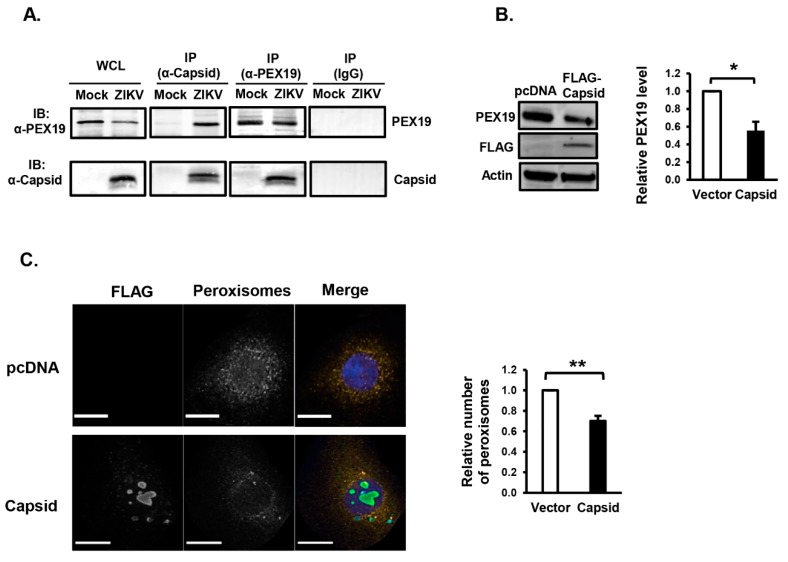
Expression of ZIKV capsid protein reduces peroxisome numbers. (**A**) U251 cells were infected with ZIKV PRVABC59 (MOI = 1). Forty-eight hours later, cell lysates were subjected to immunoprecipitation (IP) with rabbit anti-ZIKV capsid, rabbit anti-PEX19, or rabbit IgG followed by SDS-PAGE and immunoblotting (IB) with antibodies to PEX19 or ZIKV-capsid. WCL, whole-cell lysate. (**B**) HEK293T cells were transfected with a plasmid encoding FLAG-tagged ZIKV capsid or empty vector (pcDNA3.1) for 48 h. Cell lysates were subjected to SDS-PAGE and immunoblotting with antibodies to PEX19, ZIKV-capsid and actin. The relative levels of PEX19 (compared to actin) from three independent experiments were averaged and plotted. Error bars represent standard error of the mean. * *p* < 0.05. (**C**) U251 cells were transfected with a plasmid encoding FLAG-tagged ZIKV capsid or empty vector (pcDNA3.1) for 48 h and then processed for confocal microscopy. Peroxisomes were detected with a rabbit polyclonal antibody to the tri-peptide SKL and donkey anti-rabbit IgG conjugated to Alexa Fluor 546. Transfected cells expressing capsid were detected with a mouse anti-FLAG epitope antibody and donkey anti-mouse IgG conjugated to Alexa Fluor 488. Nuclei were stained using DAPI. Images were obtained using spinning disc confocal microscopy. The relative numbers of peroxisomes (SKL-positive structures) in cells transfected with or without ZIKV capsid plasmid were determined using Volocity image analysis software. Averages were calculated from three independent experiments, in which a minimum of 20 cells for each sample were analyzed. The average number of peroxisomes in mock-treated cells was normalized to 1.0. Bars represent standard error of the mean. ** *p* < 0.01.

Similar to WNV and DENV capsid proteins, the ZIKV capsid protein formed a stable complex with PEX19 (Figure 3A). Of note, interaction between ZIKV capsid and PEX19 was reported in a recent interactome study [34]. However, this was not verified by reciprocal co-immunoprecipitation or assays. To determine if the ZIKV capsid protein behaved similarly to the analogous proteins from WNV and DENV [20], U251 were transfected with a plasmid encoding FLAG-tagged ZIKV capsid or vector alone. Forty-eight hours post-transfection, cells were processed for immunoblotting or confocal microscopy. Data in Figure 3B show that the expression of ZIKV capsid protein reduced PEX19 levels by 50%, indicating that ZIKV capsid protein may be the major viral determinant that impairs peroxisome biogenesis. We next investigated peroxisome numbers in ZIKV capsid expressing cells by confocal microscopy. The expression of capsid protein was detected using anti-FLAG, and peroxisomes were identified using an antibody to the tripeptide Ser-Lys-Leu (SKL), a targeting motif found at the carboxyl termini of many peroxisomal matrix proteins [35]. The quantification of SKL-positive structures showed that transient expression of ZIKV capsid protein reduced the number of peroxisomes by 30% versus the control (Figure 3C).

### 3.3. Over-Expression of PEX11B Inhibits ZIKV Replication

**Figure 4:**
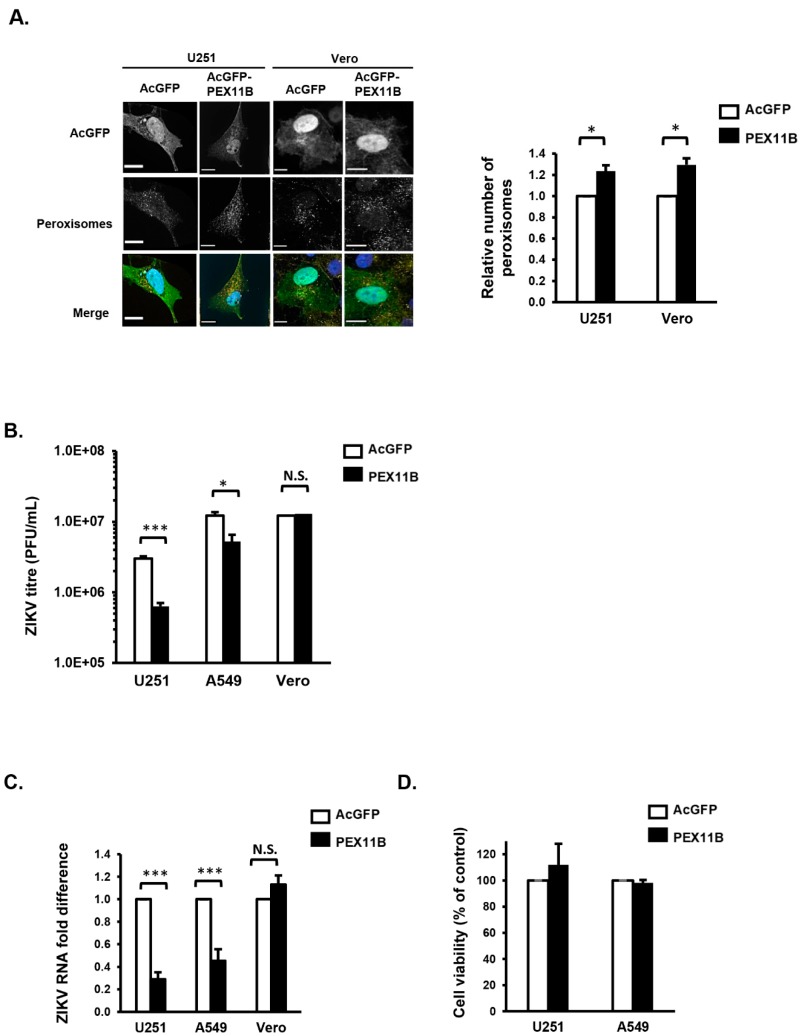
Over-expression of PEX11B inhibits ZIKV replication. (**A**) U251 or Vero cells were transduced with lentiviruses encoding AcGFP alone or AcGFP plus myc-tagged PEX11B for 48 h and then processed for confocal microscopy. Peroxisomes were detected with a rabbit polyclonal antibody to the tri-peptide SKL and donkey anti-rabbit IgG conjugated to Alexa Fluor 546. Nuclei were stained using DAPI. Images were obtained using spinning disc confocal microscopy. The relative numbers of peroxisomes (SKL-positive structures) in cells were determined using Volocity image analysis software. Averages were calculated from three independent experiments, in which a minimum of 20 cells for each sample were analyzed. The average number of peroxisomes in control cells was normalized to 1.0. Bars represent standard error of the mean. * *p* < 0.05. (**B**) U251, A549 or Vero cells were transduced with lentiviruses encoding the reporter protein AcGFP alone as a control or AcGFP plus myc-tagged PEX11B proteins for 48 h, after which the cells were infected with ZIKV PRVABC59 (MOI = 1) for another 48 h. Cell media were processed by plaque assay to determine viral titers. In parallel, cell lysates were also processed for RNA extraction and subsequent qRT-PCR to determine viral RNA level (**C**). U251 and Vero cell lysates were processed to determine cell viability (**D**). The data are averaged from the results of three independent experiments. Bars represent standard error of the mean. *** *p* < 0.001, * *p* < 0.05. N.S. = not significant.

Since flavivirus infection depletes peroxisomes, likely as a mechanism to impair the innate immune response, we questioned whether expanding the cellular pool of peroxisomes would restrict ZIKV replication. PEX11B is a cellular protein that induces peroxisome proliferation by stimulating the division of these organelles, and its over-expression results in increased numbers of peroxisomes [36,37]. U251 cells and Vero cells were transduced with lentiviruses encoding the reporter protein AcGFP alone as a control or AcGFP plus myc-tagged PEX11B for 48 h. Lentiviral-mediated over-expression of PEX11B resulted in a 20% increase in the number of peroxisomes in U251 cells (Figure 4A). 

While the effect of PEX11B expression on peroxisome proliferation was modest, the effect on ZIKV replication was dramatic. Specifically, ZIKV titers were reduced by more than 80% in U251 cells over-expressing PEX11B (Figure 4B). This effect was not limited to U251 cells as over-expression of PEX11B in A549 cells also resulted in a significant inhibition of ZIKV replication and reduction in viral titers (Figure 4B,C). To determine if the antiviral effect increasing peroxisome numbers was related to the ability of cells to mount an IFN response, PEX11B was over-expressed in Vero cells, a monkey kidney cell line which does not secrete type I IFN in response to viral infection [38]. While the over-expression of PEX11B increased the number of peroxisome in Vero cells by an average of 35% (Figure 4A), unlike in U251 or A549 cells, this peroxisome biogenesis factor did not reduce ZIKV replication or viral titers (Figure 4B,C). Data in Figure 4D confirm that the antiviral effects of PEX11B over-expression were not due to cytotoxicity in U251 or A549 cells. 

### 3.4. Over-Expression of PEX11B Enhances the Innate Immune Response 

**Figure 5:**
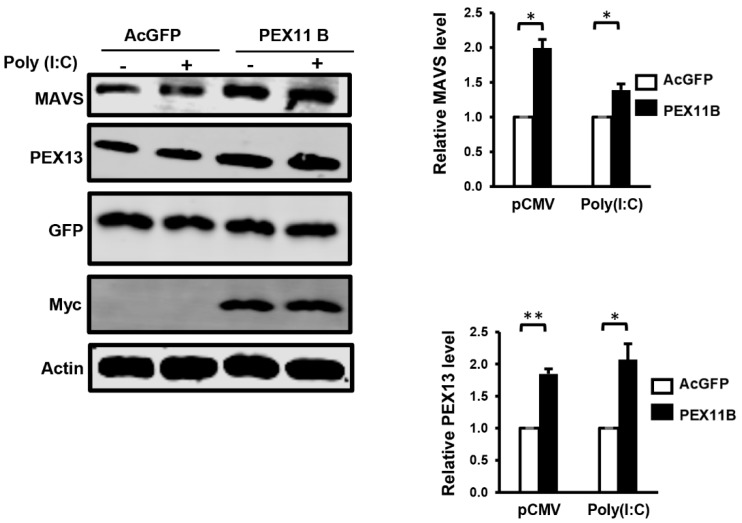
Over-expression of PEX11B increases the expression of MAVS protein. U251 cells were transduced with lentiviruses encoding AcGFP alone or AcGFP plus myc-tagged PEX11B for 48 h and then transfected with poly(I:C) (+) or an empty plasmid vector (−) for 12 h. The cell lysates were processed for immunoblot analyses with a mouse monoclonal antibody to MAVS, rabbit polyclonal PEX13, goat polyclonal antibody to GFP, and a mouse monoclonal antibody to the myc epitope. The relative levels of MAVS and PEX13 (compared to actin) from three independent experiments were averaged and plotted. Bars represent standard error of the mean. ** *p* < 0.01, * *p* < 0.05.

One possible scenario to account for the antiviral effect of PEX11B is a more robust innate immune response due to an increase in the surface area of peroxisomes, which have been termed antiviral signaling platforms [19]. However, the expansion of the peroxisome pool without a corresponding increase in the level of MAVS may not enhance antiviral signaling in response to the detection of viral genomes by RIG-I and MDA5. As such, we first investigated how the over-expression of PEX11B affected MAVS protein levels. Immunoblotting data in Figure 5 show that in cells transduced with lentiviruses encoding PEX11B, levels of MAVS protein were increased two-fold. PEX13 protein levels were also increased in response to PEX11B over-expression, suggesting that structural components of this organelle were induced by PEX11B expression. Whether the additional MAVS protein induced by PEX11B over-expression localizes exclusively to peroxisomes, mitochondria, or both is not known at this point. 

**Figure 6:**
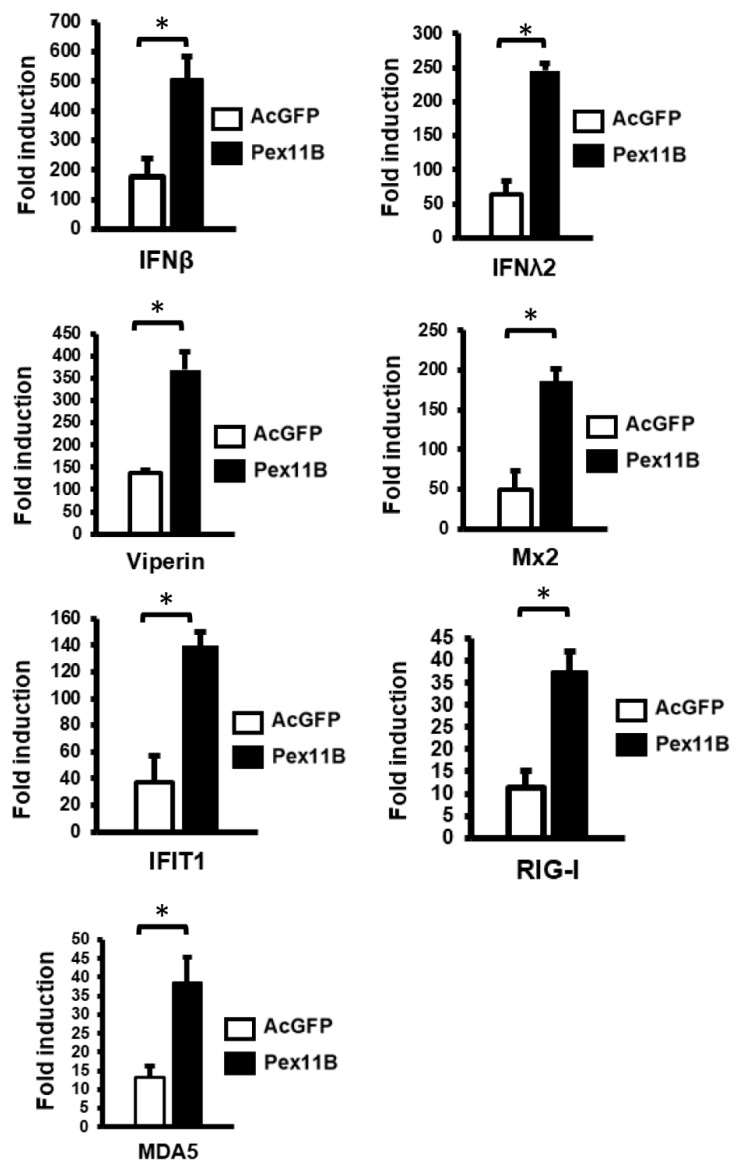
Over-expression of PEX11B enhances the innate immune response. U251 cells were transduced with lentiviruses encoding AcGFP alone or AcGFP plus myc-tagged PEX11B for 48 h and then transfected with poly(I:C) for 12 h. Cell lysates were processed for RNA extraction and subsequent qRT-PCR. Fold induction of selected ISG transcripts in response to poly(I:C) was determined. The mRNA levels of ISGs were normalized to *ACT-B* mRNA levels. The data represent the average from the results of three independent experiments. Bars represent standard error of the mean. * *p* < 0.05.

To determine if peroxisome proliferation enhanced the IFN response following the detection of viral RNA mimics such as poly(I:C), levels of type I and III IFN and ISGs were assessed in cells transduced with lentiviruses encoding PEX11B or AcGFP alone. Data in Figure 6 show that the induction of type I (*IFNβ*) and III (*IFNλ2*) IFNs was markedly elevated in cells over-expressing PEX11B. Similarly, the transcription of ISGs such as *Viperin*, *Mx2*, *IFIT1*, *RIG-I* and *MDA5* was significantly increased by PEX11B over-expression (Figure 6).

## 4. Discussion

The use of mouse models has been very useful in understanding how the IFN response affects ZIKV replication and pathogenesis. For example, whereas outbred mouse strains do not exhibit severe symptoms when infected with ZIKV, mice lacking type I IFN receptors are more susceptible to virus-induced neurological disease and death [5,6,7]. The ISG viperin has been shown to restrict the replication of many viruses in the Flaviviridae family, including Dengue, tickborne encephalitis, West Nile and hepatitis C viruses [39,40,41,42,43]. Similarly, genetic ablation of the viperin gene in mouse cells results in a more robust replication of ZIKV [44]. Of course, flaviviruses and hepaciviruses have evolved multiple strategies that are effective at blocking IFN induction and downstream signaling. Relevant examples include DENV NS2A and NS4B proteins that suppress IFN induction by inhibiting the phosphorylation of IRF3 [45,46]. During HCV infection, IFN induction can also be blocked by NS3/4A-mediated cleavage of MAVS [21]. Further downstream, the NS5 protein of DENV as well as ZIKV inhibits IFN signaling by inducing the degradation of the antiviral transcription factor STAT2 [28,47,48]. 

As well as canonical IFN induction pathways which involve RIG-I or MDA5 sensing of viral RNA followed by signaling through mitochondria-associated MAVS protein, peroxisomes are now known to play a role in IFN-based antiviral signaling [17,18,19,21,22]. A number of important pathogenic viruses have recently been shown to target peroxisomes during infection (reviewed in [49]). For instance, DENV and WNV infections result in the degradation of PEX19, which in turn leads to loss of peroxisomes and dampened induction of type III IFN [20]. Conversely, HIV infection induces the expression of miRNAs that suppress expression of multiple peroxisome biogenesis factors [23]. More recently, nsp1 protein of porcine epidemic diarrhea virus was shown to block IRF1-dependent type III IFN production by decreasing peroxisome pools, but the mechanism has yet to be elucidated [22]. 

Here, we show that ZIKV infection results in a dramatic loss of peroxisomes in primary human fetal astrocytes, a brain cell type that is highly permissive to the virus [25,50,51,52]. While we cannot rule out the potential effects of other ZIKV proteins on peroxisome biogenesis, the capsid protein appears to be the main viral determinant that causes the depletion of this organelle. The mechanism is not known but given that flavivirus capsid proteins have no enzymatic activity, they must act in concert with host cell proteins to interfere with peroxisome biogenesis and/or stability. 

As intimated above, mounting evidence suggests that peroxisome depletion may be a common facet of RNA virus infection. A consensus among multiple studies [17,18,19,20,21,22,23] is that this phenomenon is yet another strategy used by viruses to interfere with the innate immune system. In contrast, Coyaud et al. [24] reported that these organelles are important for ZIKV infection, implying that peroxisome loss is the result of being consumed or used up during the replication process. Their conclusion was based on the observation that the replication of ZIKV in fibroblast lines derived from patients with peroxisomal biogenesis disorders is lower than in fibroblasts from control patients. This must be interpreted with caution because, for ZIKV at least, we found that the permissiveness of HFAs varied significantly depending upon the individual donor [25]. This indicates that differences within the host genetic background affects ZIKV infection. Given that the genetic background of the control patients were likely different than the peroxisome biogenesis disorder patients, it cannot be ruled out that the minor differences in ZIKV replication observed were independent of peroxisomes. Moreover, at early time points and during the peak of ZIKV replication (48 h), there were no differences in titers from normal and peroxisome-deficient cells [24]. Only at 96-h post-infection was a slight decrease in viral titers observed during the infection of peroxisome-deficient cells in the study by Coyaud et al. 

While peroxisome depletion has been observed during the replication of multiple viruses, until the present study, the effects of peroxisome proliferation on viral replication had not been investigated. Our data indicate that even a modest increase in the number of peroxisomes through PEX11B over-expression results in a significant inhibition of ZIKV replication. The observation that over-expression of PEX11B in IFN-deficient Vero cells did not inhibit ZIKV replication, indicates that the enhanced antiviral response caused by peroxisome proliferation is IFN-dependent. Indeed, the induction of type I and III IFN, as well as ISGs in response to poly (I:C), was increased as much as five-fold in PEX11B-over-expressing cells. As well, levels of MAVS protein levels were two-fold higher in these cells, suggesting that the upregulation of peroxisomes is coordinated with the increased expression of antiviral signal transducing proteins. 

Together, our findings further solidify the importance of peroxisomes in antiviral defense. Moreover, the fact that peroxisome activity and abundance can be pharmacologically modulated provides compelling rationale for the investigation of peroxisome-based antiviral strategies. However, as exciting as this prospect may be, it is important to point out that in some cases, viral infection seems to increase peroxisome numbers. Specifically, it was recently reported that human cytomegalovirus upregulates the biogenesis of these organelles during infection, a scenario that enhances synthesis of plasmalogen, a peroxisome-specific phospholipid that is important for the production of nascent virions [53]. Understanding how other viruses affect these organelles will be essential as we consider the prospect of antiviral therapeutics that affect the activity or abundance of peroxisomes.

## Supplementary Materials

The following are available online at https://www.mdpi.com/2073-4409/8/7/725/s1, Table S1: Primer sets for PCR used in this study. Table S2: Primer sets for qRT-PCR used in this study.
