# Supplementary material for: Interplay between Zika Virus and Peroxisomes during Infection
Source: Cells. 2019 Jul 15;8(7):725. doi: 10.3390/cells8070725 (PMC6678468; doi:10.3390/cells8070725)
Supplement: Supplementary file 1 [file cells-08-00725-s001.pdf]

Table S1. Primer sets for PCR used in this study.

Primer name	Primer sequences (5′→3′)
3XFLAG Capsid	Forward: ATATATGCTAGCGTTTAAACGCCACCATGAAAAACCCAAAAAA GAAATCC Reverse: GGCGGGAGCGGCGGGGACTACAAAGACCATGACGGTGATTATAA AGATCATGACATCGACTACAAGGATGACGATGACAAGTAGGGCG CGCCCTCGAGATATAT
PEX11B-myc	Forward: TAGCACTAGTATGGGGAAACTG Reverse: GTACGTCGACTTACAGATCCTCTTCTGAGATGAGTTTTTGTTCGGG CTTGAGTCG

Table S2. Primer sets for qRT-PCR used in this study.

Primer name	Primer sequences (5′→3′)
Zika virus	Forward: CCTTGGATTCTTGAACGAGGA<br>Reverse: AGAGCTTCATTCTCCAGATCAA
Viperin	Forward: CTTTTGCTGGGAAGCTCTTG<br>Reverse: CAGCTGCTGCTTTCTCCTCT
MX2	Forward: CAGCCACCACCAGGAAACA<br>Reverse: TTCTGCTCGTACTGGCTGTACAG
Ifnb	Forward: TAGCACTGGCTGGAATGAGA<br>Reverse: TCCTTGGCCTTCAGGTAATG
Ifit1	Forward: AGAAGCAGGCAATCACAGAAAA<br>Reverse: CTGAAACCGACCATAGTGGAAAT
Actb	Forward: CCTGGCACCCAGCACAAT<br>Reverse: GCCGATCCACACGGAGTACT
IFN-λ2	Forward: AGTTCCGGGCCTGTATCCAG<br>Reverse: GAACCGGTACAGCCAATGGT
RIG-I	Forward: AGTGAGCATGCACGAATGAA<br>Reverse: GGGATCCCTGGAAACACTTT
MDA5	Forward: GAGCAACTTCTTTCAACCACAG<br>Reverse: CACTTCCTTCTGCCAAACTTG
